# Application of iron nanaoparticles in landfill leachate treatment - case study: Hamadan landfill leachate

**DOI:** 10.1186/1735-2746-9-36

**Published:** 2012-12-27

**Authors:** Zahra Esfahani Kashitarash, Samadi Mohammad Taghi, Naddafi Kazem, Afkhami Abbass, Rahmani Alireza

**Affiliations:** 1 University of Applied Science and Technology, Water & Wastewater Engineering Department, Power & Water University of Technology, Tehran, Iran; 2 Department of Environmental Health Engineering, Faculty of Health and Research Center for Health Sciences, Hamadan University of Medical Sciences, Hamadan, Iran; 3 Department of Environmental Health Engineering, School of Public Health, Tehran University of Medical Sciences, Tehran, Iran; 4 Faculty of Chemistry, Bu-Ali Sina University, Hamadan, Iran

**Keywords:** Leachate treatment, Iron nanoparticles, Landfill, Hamadan

## Abstract

This study was performed with the objective of determining the efficiency of iron nanoparticles for reducing chemical oxygen demand (COD), 5-day biological oxygen demand (BOD_5_), total solids (TS) and color of Hamadan city landfill leachate. Experiments were performed in a batch reactor and the main effective factors of pH, reaction time and concentration of iron nanoparticles were investigated. The obtained data were analyzed with One-Way ANOVA statistical test and SPSS-13 software. Maximum removal efficiencies were 47.94%, 35%, 55.62% and 76.66% for COD, BOD_5_, TS and color, respectively (for 2.5 g/L iron nanoparticles dosage, pH = 6.5 and 10 min reaction time). The results showed that the removal of COD, BOD_5_ and color had reverse relationship with contact time and TS removal followed a direct relationship (P < 0.05). Iron nanoparticles could remove averagely 53% of leachate COD, BOD_5_, TS and color in a short contact time (10 min) increasing pH up to 6.5, increased the removal efficiency for COD, BOD_5_, TS and color and then removal efficiency decreased with increasing pH to 8.5. Increasing the dosage of nanoparticles to 2.5 g/L increased the efficiency of process. High compatibility and efficiency of this process was proven by landfill leachate pre-treatment or post-treatment, so this removal method may be recommended for municipal solid waste landfill leachate treatment plants.

## Introduction

Landfill leachate is a very strong wastewater and has created many environmental concerns due to a wide variety of pollutants. Leachate is a malodorous liquid with dark-brown color that has to leak out from inside wastes and involves many types of organic materials in the form of suspended and dissolved, pathogenic agents and toxic compounds such as heavy metals and heavy organic materials [1-3]. Leachate penetrates into the soil and reaching the underground water table, causes pollution of soil and water. It can move horizontally in municipal landfill sites and cause surface water pollution and if the water is used by the public, the probability of outbreak of dangerous diseases is high [2].

Different methods such as leachate recirculation into vessels of landfill (in site treatment), leachate evaporation, discharge to sewage treatment plants, and treatment of leachate by physicochemical and biological methods have been used for management of leachate [1-4]. Pre-treatment or complete treatment of leachate is required where leachate evaporation and recirculation cannot be used, or direct disposal of leachate to wastewater treatment facilities is not possible [4,5].

Nanoparticles due to small size, high surface area, crystal form, unique network order, and highly reactivity can be used for purification and treatment of pollutants to non hazardous materials [6,7]. Small size of iron nanoparticles makes easy and effective subsurface distribution, as while their large cross-section makes high reactivity and rapid destruction of the pollutants. There are many reports about the use of nanoparticles as a purification process for removal pollutants from the environment. Researches indicate that nanoparticles of iron can act as a reducing agent and catalyst in detoxification of a large number of pollutants in the environment, such as organic pesticides, Trinitrotoluene (TNT), heavy metals, nitrates, polychlorinated biphenyls (PCBs) [6-8]. Iron nanoparticles are considered as a novel technology that are effective to transfer and detoxification a wide variety of environmental pollutants such as chlorinated solvents, and organic contaminants [8,9].

In this study the efficiency of iron nanoparticles was investigated to reduce the concentrations of COD, BOD_5_, TS and color of Hamadan municipal solid waste (MSW) landfill leachate located in the west of Iran. Also the effect of pH, reaction time and nZVI dosage variation were investigated to determine the optimum conditions.

## Materials and methods

A pilot was designed and performed at a batch system to investigate COD, BOD_5_, TS and color removal of Hamadan landfill leachate using iron nanoparticles (nanoparticles characteristics represented in Table 1).

**Table 1:** **Physico**-**chemical properties of used iron nanoparticles in this study**

**Properties**	**Average value**
Specific surface area (BET), m^2^/g	>12
Average primary particle size, nm	30-60
Particle full range, nm	5-200
Particles^,^ shape	Spherical
Fe-state	Ferromagnetic
Bulk density, g/cm^3^	0 > 0.5
Carbon content, wt%	11-14
Carbon admixture, %	

### Landfill leachate characteristics

Hamadan landfill is located in the west of Iran. Leachate is generated from about 750 tons/day of municipal solid waste and 5 tons/day of hospital wastes disposed in this place. There wasn’t any treatment facility in this field and raw leachate is discharged into the environment without any treatment. Properties of Hamadan Leachate are presented in Table 2.

**Table 2:** Properties of Hamadan landfill raw leachate

**Parameter**	**Range**
BOD_5_, mg/L	20000
COD, mg/L	85000
TS, mg/L	200000
Color, Pt.Co	15000
pH	6.5
BOD_5_/COD	0.34

### Sampling

Leachate samples were collected from Hamadan landfill in spring season in polyethylene bottles and were kept at temperatures of 4°C in order to avoid the sample parameters variations [10]. The site of sampling was selected in a way that samples could represent the whole landfill leachate conditions.

### Analytical methods and chemical agents

Chemical oxygen demand (COD), 5-day biochemical oxygen demand (BOD_5_), total solids (TS) were determined according to standard methods [10]. Color was determined by 8025 HACH DR. 2500. Iron nanoparticles were prepared from Plasmachem. GmbH Berlin, Germany, and other chemicals were purchased from Merck Company, Germany.

### Batch experiments

For the experiments performed in a batch reactor, the most effective factors as pH (2.5, 4.5, 6.5 and 8.5), reaction time (5, 10, 15 and 20 min) and concentration of iron nanoparticles (1250, 2500, 4000 and 5000 mg/L) were investigated. Analysis of samples in each step was triplicated to increase the reliability, and accuracy and precision of the experiments.

Removal efficiency (E) at different stages was calculated by using the results and the initial concentrations with the equation (1):

(1) $$E=\frac{C_{i}-C_{f}}{C_{i}}\times 100$$

where C_i_ and C_f_ are the initial concentration and final concentration of studied parameters, respectively.

### Statistical tests

The optimum values of each variable were selected and the performance of iron nanoparticles was evaluated by using One-Way ANOVA test statistic and SPSS-13 software.

## Results

**Figure 1:**
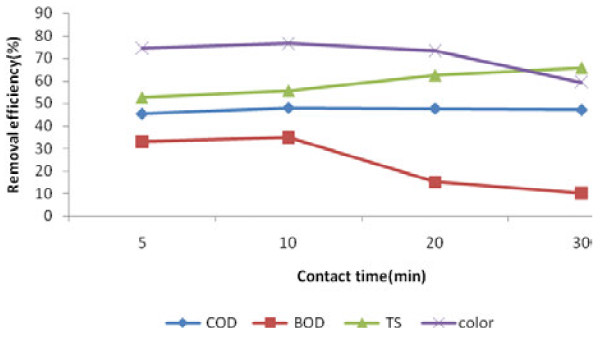
Effect of contact time variation on the removal efficiency of COD, BOD_5_, TS and color of leachate (T = 18 ± 1°C, pH = 6.5, iron nanoparticles concentration = 2500 mg/L).

**Figure 2:**
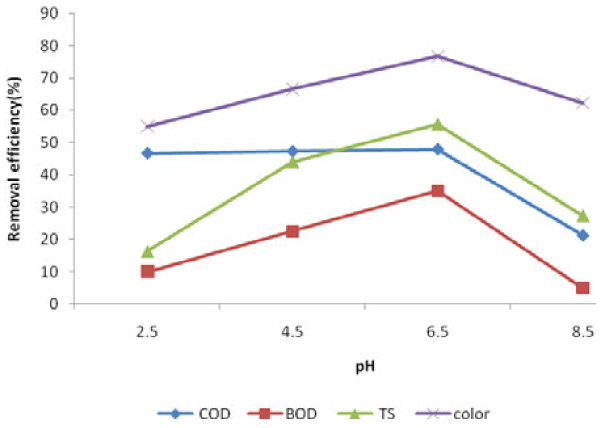
Effect of pH on the leachate COD, BOD_5_, TS and color removal efficiency (T = 18 ± 1°C, iron nanoparticles concentration = 2500 mg/L, reaction time = 10 min).

**Figure 3:**
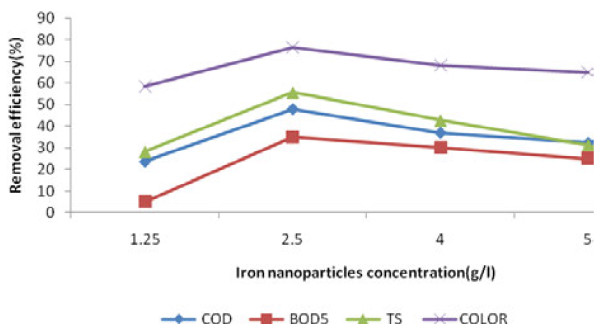
Effect of iron nanoparticles variation on COD, BOD_5_, TS and color removal efficiency of leachate (T = 18 ± 1°C, pH = 6.5, reaction time = 10 min).

The results of contact time, pH and dosage of iron nanoparticles variations on BOD_5_, COD, TS and color removal efficiency are presented in Figures 1, 2 and 3.

## Discussion

### The effect of contact time variation

As shown in Figure 1 COD removal efficiency at concentration of 2500 mg/L of nanoparticles decreased from 47.94% to 47.05% with increasing contact time to 30 min and BOD_5_ removal efficiency dropped from 35% to 10%. As for color, results were 76.66% to 59.33%, while TS removal efficiency increased from 55.62% to 65.85%. Analysis performed by One-Way ANOVA test, showed significant differences between the various contact times (p-value<0.05 for BOD_5_, COD, TS and color). High efficiency of COD, BOD_5_ and color removal at 10 min indicates the high capability of iron nanoparticles in attracting organic materials existing in the leachate, but TS need higher contact times (30 min.). Increasing the pitting and corrosion on the surface of iron nanoparticles with time and therefore absorption cross sections are the basic reasons of increasing COD, BOD5, TS and color removal efficiency. For contact time > 10 min, the removal efficiency decreased and created an opportunity for iron to react with oxygen and producing complexes of Fe (III),(especially in high pH). Also the deposits of iron were yellow and decreasing in color efficiency will be accured after 10 min. Moreover as shown in Figure 1, TS efficiency increased when contact time increased to 30 min, because more reaction has been taken place in higher contact time. It has been demonstrated that arsenate concentration can reach to zero from 1 mg/L in 10 min with concentration of 0.1 g/L iron nanoparticles [11]. Also the studies on color removal of Acid Black 14 by iron nanoparticles determined that the removal efficiencies in 2 min, 15 min and 30 min were 30%, 70%and 74%, respectively [12].

### The effect of pH variation

Analysis of obtained removal efficiency of the COD, BOD_5_, TS and color in various pH levels by One-Way ANOVA test showed a significant difference (p< 0.05). It means that changes in pH can change the removal efficiency of COD, BOD_5_, TS and color. Increasing of the pH value up to 6.5, increased the removal efficiency and then it decreased with increasing pH up to 8.5 (Figure 2). Removal efficiencies decrease were 26.6%, 30%, 28.4% and 50.3% for COD, BOD5, TS and TS, respectively. Based on a study results, the ferrous ions dissolve in alkaline conditions due to contact of nanoparticles surface with hydroxyl radicals can cause the production of ferrous hydroxide precipitation. This deposit will occupy the active surface sites on nanoparticles and prevent more activity [12]. Also results of Kanel research showed that absorption of arsenic on nanoparticles is 100% in pH = 3-7. However, this absorption reached 84/7% at pH = 9 and 37/9% at pH = 11. Therefore, nanoparticles are more effective in acidic pH than alkaline pH [11].

The three major reasons for the difference between results of similar studies and this study (that in pH close to the 7 have been more effective) were: 1) before iron nanoparticles could act as an adsorbent it may be resolved in acidic pH and outside the system and hence cannot react with pollutants; 2) pH can be also effective on reaction rate. In other word, reaction rate of iron nanoparticles with pollutants could reduced in acidic pH; 3) pollutants were in a form that couldn’t be highly adsorbed in acidic pH and the best results were achieved in almost neutral pH (pH = 6.5). In a research on humic acid removal by iron nanoparticles,the highest amount of humic acid attracted by iron nanoparticles has been achieved at pH = 3-9 and removal efficiency decreased quickly with increasing pH to more than 10. The researchers believed that nanoparticles of iron were positively charged with decreasing pH and negatively charged with increasing pH. So, negatively charged humic acid adsorption increased with decreasing pH [13].

These results indicated that iron nanoparticles are able to operate in the range of Hamadan landfill leachate pH. Therefore, it can be consideration as economically efficient option for leachate pre-treatment and also can be used to change BOD_5_/COD ratio for biological treatment process, as subsequent treatment (because BOD_5_/COD ratio reached 0.73 from 0.34 after treating by iron nanoparticles). It indicates that an iron nanoparticle has increased the ability of biological treatment of leachate.

### The effect of iron nanoparticles concentration variation

The effects of iron nanoparticles dosage on BOD_5_, COD, TS and color removal efficiency of Hamadan landfill leachate is presented in Figure 3 . One-way ANOVA test showed a significant difference between various concentrations of nanoparticles for BOD_5_, COD, TS and color (p<0.05). It means that different concentrations of iron nanoparticles were effective for the removal efficiency. Hence, the removal efficiency increased with increasing the concentration of nanoparticles from 1250 mg/L to 2500 mg/L. These results indicate that iron nanoparticles can remove averagely 53% of leachate BOD_5_, COD, TS and color in a short contact time (10 min). Increasing of the adsorption active sites and thus the possibility of more dealing of leachate inorganic and organic materials with iron nanoparticles and also increasing oxidation and reduction reactions were the main causes of increasing removal efficiency with increasing concentration of nanoparticles [14].

It is essential to note that increasing of iron nanoparticles concentration from 2500 mg/L to 4000 mg/L and 5000 mg/L reduced removal efficiency in mentioned conditions. These results showed that the excess amounts of iron nanoparticles can cause turbidity, and interference in the leachate treatment. As a result, treatment efficiency would be reduced. A research on azure color removal with five concentrations of iron nanoparticles has shown that high concentrations of iron nanoparticles decreased azo color residuals, so that the removal rate of color was more than 95% and less than 15% in the concentrations of 0.3348 g/L and 0.0335 g/L of nanoparticles, respectively [12]. In other similar research, removal efficiency of hexavalent chromium by iron nanoparticles was 100% in concentration of 0.4 g/L nanoparticles, and has decreased to 26% in 0.1 g/L nanoparticles. Obtained results of a study concluded that 0.1 g/L concentration of nanoparticles couldn’t provide enough active surface sites for removal of hexavalent chromium [15].

## Conclusion

The effect of Nanosized Zero Valent Iron (NZVI) for treatment of Hamadan landfill leachate (initial COD = 85000 mg/L) was investigated in this paper. It was observed this technique was a fast procedure and 47.94% removal efficiency was obtained in 10 min. The optimal condition was at pH value about 6.5, temperature of 18 ± 1°C and iron nanoparticles concentration of 2500 mg/L. High compatibility and efficiency of this process was proven by landfill leachate pre-treatment or post-treatment, so this removal method may be recommended for municipal solid waste landfill leachate treatment plants.

## Competing interests

The authors declare that they have no competing interests.

## Authors’ contributions

All authors contributed equally to this work. KEZ designed and performed experiments, analysed data and wrote the paper. SMT conceived the strategies, developed the concept, supervised the project and wrote the Supplementary Information. NK and AA and RAR designed the study, gave technical support and conceptual advice. All authors discussed the results and implications and commented on the manuscript at all stages. All authors read and approved the final manuscript.
